# Monitoring Thermal Ablation via Microwave Tomography: An Ex Vivo Experimental Assessment

**DOI:** 10.3390/diagnostics8040081

**Published:** 2018-12-06

**Authors:** Rosa Scapaticci, Vanni Lopresto, Rosanna Pinto, Marta Cavagnaro, Lorenzo Crocco

**Affiliations:** 1 National Research Council of Italy—Institute for the Electromagnetic Sensing of the Environment, 80124 Napoli, Italy; crocco.l@irea.cnr.it; 2 Italian National Agency for New Technologies, Energy and Sustainable Economic Development, Division of Health Protection Technologies, Casaccia Research Center, 00123 Rome, Italy; vanni.lopresto@enea.it (V.L.); rosanna.pinto@enea.it (R.P.); 3 Department of Information Engineering, Electronics and Telecommunications, Sapienza University of Rome, 00184 Rome, Italy; marta.cavagnaro@uniroma1.it

**Keywords:** microwave imaging, thermal ablation, microwave ablation, image-guided, monitoring, dielectric properties

## Abstract

Thermal ablation treatments are gaining a lot of attention in the clinics thanks to their reduced invasiveness and their capability of treating non-surgical patients. The effectiveness of these treatments and their impact in the hospital’s routine would significantly increase if paired with a monitoring technique able to control the evolution of the treated area in real-time. This is particularly relevant in microwave thermal ablation, wherein the capability of treating larger tumors in a shorter time needs proper monitoring. Current diagnostic imaging techniques do not provide effective solutions to this issue for a number of reasons, including economical sustainability and safety. Hence, the development of alternative modalities is of interest. Microwave tomography, which aims at imaging the electromagnetic properties of a target under test, has been recently proposed for this scope, given the significant temperature-dependent changes of the dielectric properties of human tissues induced by thermal ablation. In this paper, the outcomes of the first *ex vivo* experimental study, performed to assess the expected potentialities of microwave tomography, are presented. The paper describes the validation study dealing with the imaging of the changes occurring in thermal ablation treatments. The experimental test was carried out on two *ex vivo* bovine liver samples and the reported results show the capability of microwave tomography of imaging the transition between ablated and untreated tissue. Moreover, the discussion section provides some guidelines to follow in order to improve the achievable performances.

## 1. Introduction

Thermal ablation is a therapeutic procedure used to destroy unhealthy tissue by way of a very high and localized temperature increase. In thermal ablation, the target temperature is close to 60 °C in the zone of ablation, which for tumor treatment should include the pathologic lesion plus a 5−10 mm safety margin of healthy tissue [1,2]. At this temperature, an almost instantaneous cell death by way of coagulative necrosis is achieved [1].

The increase in temperature can be obtained using different energy sources, such as radiofrequency currents, ultrasounds, lasers [3]. Among the others, microwave thermal ablation (MTA), in which the energy source is an electromagnetic field in the Industrial, Scientific and Medical (ISM) frequency band (typically at 915 MHz or 2.45 GHz), is gaining an increasing attention in the clinical practice [4,5], owing to its capability of treating larger tumors in a shorter time with respect to other ablation modalities [2]. As a matter of fact, MTA is increasingly used to treat different types of solid tumors, as those of the liver, kidney, lung, etc. [6,7,8,9,10].

The MTA clinical set-up is typically made by a minimally invasive interstitial applicator (i.e., a microwave ablation antenna), whose diameter is in the order of a few mm, a microwave power generator, and a cooling system used to keep the antenna’s shaft at safe temperatures. The clinical procedure foresees the introduction of the antenna into the patient’s body, percutaneously or following natural paths, and the onset of the microwave generator with a power value and for a time duration depending on the dimension of the tumor to be treated (typically, 60–100 W for about 5−10 min) [4,5]. Commercial systems give coagulative performances of the devices based on experiments performed either *ex vivo* or in vivo on animals [11,12]. Clinicians use these data to define the clinical protocol, i.e., the power value and time of irradiation to be used in a defined pathological situation. Moreover, software tools have been recently developed to help defining the best insertion path for the antenna [13].

Before the treatment, to help targeting the applicator in the center of the tumor to be treated, clinicians use image-guidance techniques such as ultrasounds (US), computerized tomography (CT), or magnetic resonance imaging (MRI). During the treatment, temperature is monitored by temperature sensors (usually thermocouples), whose positions are carefully chosen to assure safe temperatures in critical organs close to the tumor to be treated [14,15,16,17,18,19,20,21,22,23,24]. Techniques which could be used to monitor the evolution of the thermally ablated area during the treatment include US, CT, and MRI. However, all these techniques show drawbacks, which prevent their integration into MTA systems. In particular, US would be the most natural choice for MTA real-time monitoring, due to its widespread availability, low cost, and real-time imaging up to sub-millimeter resolutions [17]. However, US can be scarcely effective for the real-time monitoring of MTA procedures, because it is blinded by a hyper-echogenic cloud caused by water vaporization in the heated tissue, which conceals the applicator and the tumor [1,18].

With reference to CT, studies investigated the best sequences to be used for real-time thermometry of radiofrequency thermal ablation [19,20,21]. The contra-indications of CT are mainly related to the exposure of the patient and the clinician to the ionizing radiation of CT, with a dose that depends on the duration of the MTA procedure and on the number of performed scans. Moreover, CT scanners do not have real-time capabilities, and perform fixed imaging in the axial plane, which leads to difficulties in treatments to be performed under the diaphragm or in other areas where oblique imaging planes are desirable [17].

MRI is potentially the most accurate and safe technique to perform real-time thermometry during the procedure [14,23], since temperature can be obtained from T1 relaxation time or proton resonance frequency (PRF) shift [24]. However, MRI use is limited by technical difficulties related to (unavoidable) motion artefacts, electromagnetic compatibility issues with the microwave antenna, and, last but not least, the high cost of the MRI equipment, which entails a significant impact in terms of economical sustainability for health systems [18].

Accordingly, the lack of a reliable, low-cost, real-time imaging system represents a weak point of thermal ablation procedures, especially those using microwave power, thus impairing their widespread use in the clinics [16]. For this reason, research is pushing towards the development of a non-invasive real-time monitoring system, both trying to improve existing techniques and looking for brand new solutions.

Microwave tomography (MWT) has been recently proposed as an alternative imaging modality for non-invasive real-time monitoring of thermal ablation procedures [25,26,27]. MWT images the variation of the electromagnetic properties with respect to an unperturbed situation, by recording (and properly processing) the electromagnetic field backscattered by the region of interest when probed by a known incident wave. Given the experimentally observed evidence that tissues undergo dramatic changes during ablation treatments [28,29,30], MWT is in principle viable for thermal ablation monitoring. Moreover, MWT involves low-cost and portable equipment as it exploits standard components—such as microwave (MW) antennas, MW generators, amplifiers—whose size and cost have considerably reduced in the last years thanks to the progress in the field of telecommunications. Finally, MWT is completely harmless, being based on the use of low-power non-ionizing radiations.

The basic principle of MWT thermal ablation monitoring is to probe the treated region with an array of antennas, external to the patient body, and record the evolution of the back-scattered field during the treatment. The variations of the recorded data between different time instants are then processed by means of a suitable inverse scattering algorithm, whose output is an image of the changes occurring in the electromagnetic properties of the scenario under test. In particular, to enable real-time operations, linearized inversion models can be exploited, based on the circumstance than only localized variations occur during the treatment and that the main (or first) clinical goal is to detect the boundary between treated and untreated tissue.

The potential of MWT monitored thermal ablation has been so far investigated in silico. In particular, Scapaticci et al. [27] showed the possibility of imaging the evolution of thermal ablation within a sample of liver tissue, whereas [31] simulated the monitoring of an interstitial heating procedure of a brain tumor. In this paper, the first experimental proof-of-concept of thermal ablation monitoring via MWT is reported. In particular, the results from two MTA procedures carried out on *ex vivo* liver tissue are described and discussed. The tomographic approach is the same as the one assessed in the previous in silico study [27], properly adapted to the measurement configuration adopted in the experiments. In particular, the changes occurring in the samples before and after microwave ablation are imaged, with the aim of appraising the boundary between treated and untreated tissue.

The paper is structured as follows. In the next section, the adopted material and methods are described. In particular, the experimental set-up developed for the validation is described along with the protocol adopted for thermal ablation. Then, the MWT algorithm is recalled and particularized to the adopted configuration. The results are presented in the subsequent section, preceded by the visual analysis of the ablated specimens, which provides the information necessary to assess the imaging outcomes. Discussion and conclusions follow.

## 2. Materials and Methods

### 2.1. Experimental Set-Up

**Figure 1:**
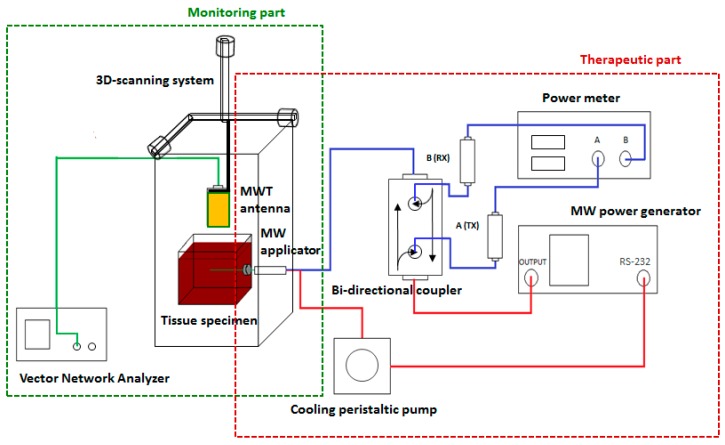
The experimental setup developed for the proof of concept. Tx and Rx denote part of the transmitted signal (Tx) and part of the reflected one (Rx) collected by the directional coupler to measure the actual power fed to the MTA antenna. The green line represents the connection between the vector network analyzed (VNA) and the MWT antenna. The red lines represent the connection of the MW power generator with the bidirectional coupler and with the peristaltic pump, and of the peristaltic pump with the MW applicator. The blue lines represent the connections of the power meter with the bidirectional coupler, and of the bidirectional coupler with the MW applicator. The black arrows refer to conventional symbols used for bidirectional coupler, which identify the directions of propagation of the direct power (forward) and of the reflected power (backward).

The conceptual scheme of the experimental setup developed for the present proof-of-concept experiments is depicted in Figure 1. The setup consisted of two main parts: the ‘therapeutic’ one, on the right side of the picture, which was in charge of performing MTA, and the ‘monitoring’ part, on the left side, which gathered the data required for the MWT processing.

The MTA subsystem was based on a commercial ablation apparatus (HS AMICA, HS Hospital Service S.p.A., Rome, Italy), consisting of a programmable microwave power generator (available power: 100 W continuous wave (CW), frequency: 2.45 GHz), connected through a coaxial cable to a 14-gauge cooled-shaft percutaneous applicator. The MTA applicator was a coaxial dipole antenna. The antenna was equipped with a mini-choke to confine the energy emission in the zone to be treated [32]. The applicator was cooled by means of water pumped by a peristaltic pump (at a constant velocity of 40 mL/min), circulating into the shaft up to the mini-choke section.

The MW power, fed to and reflected from the applicator, was monitored by a two-channel digital power meter (Agilent E4419B, Agilent Technologies Inc., Santa Clara, CA, USA) and a Type-N dual-coaxial reflectometer coupler (Narda 3022, Narda Microwave Corp., Hauppauge, NY, USA).

**Figure 2:**
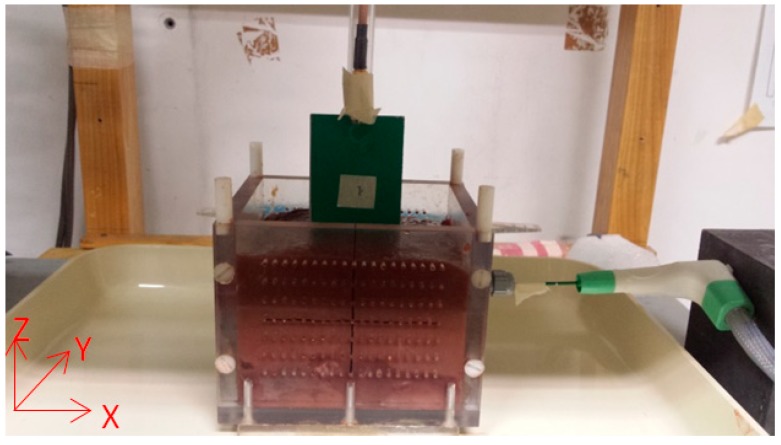
Plastic box containing an *ex vivo* tissue specimen of bovine liver. The MWT antenna is visible on the top of the specimen connected to the arm of the scanning system. The MTA applicator is visible on the right of the specimen, partially inserted into it. The reference system is shown on the bottom left in red color.

MTA was performed on specimens of *ex vivo* bovine liver taken from a slaughter house. A box of polymethyl-methacrylate (PMMA)—a material typically with negligible losses and low dielectric constant (e.g., about 2.9) [33], i.e., almost transparent to MW fields—with internal dimensions of 120 × 100 × 100 mm^3^, was used to hold the tissue specimens (size 120 × 100 × 80 mm^3^) and to allow an accurate and repeatable insertion of the MTA applicator (Figure 2). Specifically, the MTA applicator was introduced in the specimen through a hole located at the center of the front-side wall of the box, along the *x*-axis (see Figure 2), so that the distal tip of the applicator was inserted into the tissue specimen at a depth of about 7 cm, and the feed was approximately located in correspondence of the barycenter of the tissue specimen (Figure 2).

The MWT subsystem consisted of a microwave antenna connected to a vector network analyzer (VNA, Keysight E5071C ENA, 9 kHz–4.5 GHz, Keysight Technologies, Santa Clara, CA, USA) measuring the reflection coefficient (S_11_, magnitude and phase). A multi-monostatic acquisition was performed moving the antenna along a rectilinear path (oriented along the *y*-axis) above the specimen surface (Figure 2), by means of a remotely controlled three-dimensional (3D) scanning system with 0.1-mm spatial resolution (ITALMETRON, Rome, Italy). The scanning system was controlled by a purposely developed routine in Labview^TM^.

### 2.2. MTA Experiments

The MTA experiments were performed on two different *ex vivo* tissue specimens of bovine liver. In both cases, an average net power of about 60 W at 2.45 GHz (CW) for a time of 8 min was delivered to the applicator. The ablation protocol (power and time) was chosen in such a way to achieve an ablated zone completely included in the specimen, with a margin of untreated tissue between the boundary of the ablation and the surface of the specimen [34].

In order to assess the outcomes of the imaging procedure, the algorithm’s results were compared to the actual scenario. To obtain a description of such a ‘ground truth’ a visual inspection of the zone of ablation was performed. To this end, at the end of the MTA procedure, the specimen was sectioned. In particular, the specimen was cut in the *xy* plane (see Figure 2), at a depth corresponding to the height at which the applicator was inserted. In the *xy* plane, the zone of ablation achieved with the considered cooled-shaft applicator typically consists in an ellipsoidal-shaped thermally coagulated area of ablated-but-not-carbonized tissue encompassing an arrow-shaped central region of carbonized tissue [29]. In the transversal plane—i.e., the plane orthogonal to the shaft of the applicator (*yz* in Figure 2)—the thermal lesion has typically a circular shape with a rim of white coagulated tissue surrounding the central carbonized area [29].

The characteristic dimensions of the ablated zone, defined in terms of maximum extension in the longitudinal (i.e., parallel to the shaft of the applicator, *x*-axis in Figure 2) and transverse (*y*-axis in Figure 2) directions, were measured with a ruler (accuracy ±0.5 mm). Likewise, the maximum extension of the central carbonized region was measured in the longitudinal and transverse directions. Moreover, the height of the specimen in the antero-posterior direction (*z*-axis in Figure 2) was measured prior and after completion of the ablation procedure, to assess possible deformation of the specimen linked to ablation-induced tissue modifications [34,35]. The distance between the upper boundary of the ablated zone and the surface of the specimen was measured post-ablation to assess the extension in the antero-posterior direction of the margin of untreated tissue.

### 2.3. MWT Measurements

For each MTA experiment, MWT measurements (S_11_, magnitude and phase) were performed in two different conditions, i.e., pre-ablation (untreated tissue) and right after completion of the MTA procedure. The resulting differential scattering parameters provide the data required for the MWT processing, since the changes in the dielectric properties of the specimen due to the ablation are expected to be reflected by the variations of the scattering coefficients [28].

**Figure 3:**
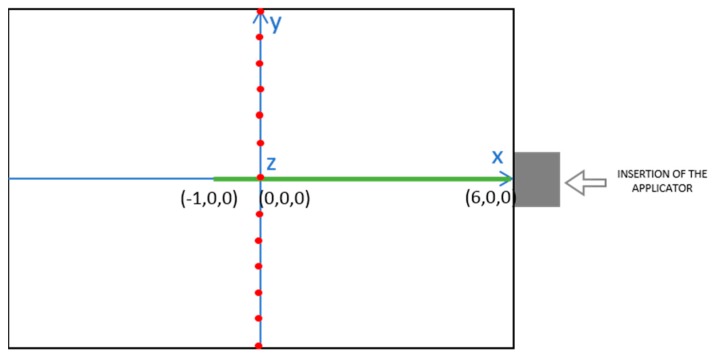
Positioning of the MTA applicator in the tissue specimen (green line) and MWT measurement points (red dots). View on a coronal plane at a height of about 40 mm from the applicator. Distances are expressed in cm. The blue lines denote the coordinate axes, while grey square and white arrow indicate the applicator and its insertion direction, respectively.

MWT measurements were carried out in the 1–4 GHz frequency band (201 frequency points) at 13 evenly spaced positions along the *y*-axis, in correspondence of the applicator’s feed (*x* = 0). In particular, the antenna was moved from *y* = −30 mm to *y* = +30 mm with a spatial step of 5 mm, as shown in Figure 3 (red dots in the figure). It is to be noted that both the measurements performed before and those performed after ablation were conducted with the applicator inserted into the specimen, but turned off.

For each experiment, MWT measurements were carried out for two heights of the antenna. In particular, measurements were taken with the antenna in contact with the surface of the specimen, that is at *z* = 30 mm, putting the origin (*z* = 0) in correspondence to the shaft of the applicator, and with the antenna at a height *z* = 40 mm. For this latter case, considering the transverse dimension of the tissue specimen (height of about 8 cm) and the insertion position of the applicator (at the height of about 5 cm from the bottom of the specimen) and by neglecting not predictable ablation-induced tissue deformation [34,35], this corresponds to an average distance of about 10 mm between the distal edge of the MWT antenna and the surface of the tissue specimen.

**Figure 4:**
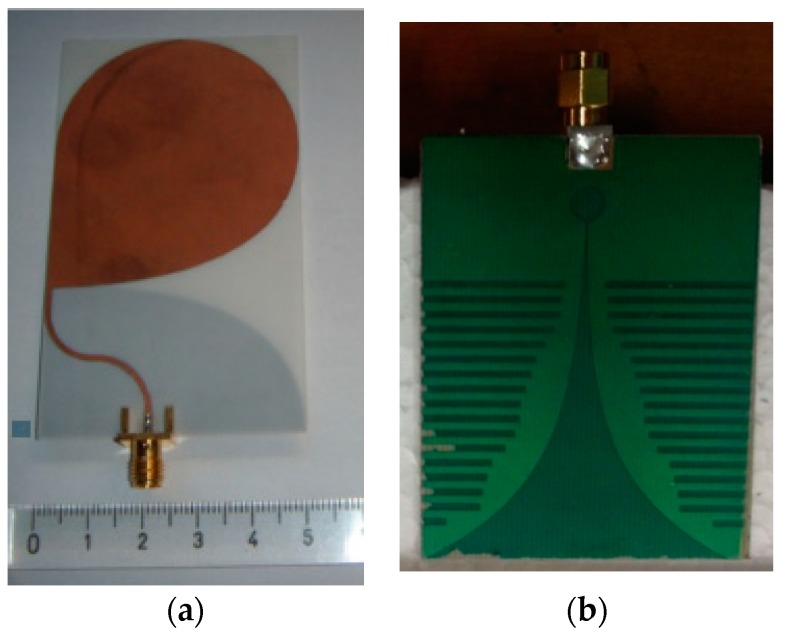
MWT antennas: (**a**) half-heart shape; (**b**) Vivaldi shape.

In order to make fully independent experiments, the two MTA procedures were monitored employing two different ultra-wide band antennas. The first antenna (Figure 4a) consisted in a coplanar antipodal configuration with a ‘half-heart’ geometry (dimension 50 × 85 mm^2^) printed on a Rogers substrate (RO4003, relative permittivity *ε_r_* = 3.38) [36]. The second antenna (Figure 4b) is a coplanar Vivaldi configuration (dimension 54 × 68 mm^2^) printed on a Taconic substrate (RF-35, *ε_r_* = 3.50) [37].

It is worth noting that the two antennas were not specifically designed for the purpose of this experiment. Nevertheless, see Appendix A, they showed an acceptable behavior throughout the measurement bandwidth, when operated in presence of the specimen, in particular when they were not in contact with it (as expected, given the fact they were not optimized).

### 2.4. Imaging Algorithm and Assessment Criterion

To form the image of the monitored ablation scenario, a differential microwave tomography approach based on the Born approximation was adopted, similar to the one considered in previous studies [28,29]. Such an approach allows to image (in a qualitative fashion) the variations occurring in the scenario under test, which was indeed the scope of this initial experimental study. Notably, the approach uses the truncated singular value decomposition (TSVD) algorithm [38], which allows real-time results, since its computationally intensive part can be run off-line before data acquisition. In the following, the TSVD inversion algorithm, as particularized for the experimental configuration, is recalled, whereas the formulation details are given in Appendix B.

The experimental data were acquired along a rectilinear domain, hence, the available information was not adequate for imaging a 3D region. Accordingly, a 2D domain corresponding to the cross section of the specimen along the *yz* plane, i.e., orthogonal the applicator shaft, in correspondence of the line of scan, was taken as region of interest Ω. Such a domain was then discretized into $P=N_{y}\times N_{z}$ square pixels of 0.5 mm resolution. In particular, $N_{y}=201$, and $N_{z}=79$ when the antenna is at *z* = 30 mm and $N_{z}=99$ when the antenna is at *z* = 40 mm.

In the TSVD scheme, the unknown vector is directly retrieved by applying the inversion formula to the data vector ΔS:

(1) $$\Delta \chi =K_{R}^{+}\Delta S$$

where Δχ denotes the P×1 column vector of the unknowns (the 2D matrix encoding the contrast is rearranged into a 1D vector for the sake of implementation of the TSVD algorithm), whose generic element encodes the differential contrast value, assumed non-dispersive, in the relevant pixel, defined as

(2) $$\Delta \chi_{j}=\frac{\Delta \varepsilon_{j}}{\varepsilon_{L}},\;j=1,\ldots ,\;P$$

where $\Delta \varepsilon_{j}$ is the variation of the complex permittivity in the *j*-th pixel due to the ablation profile and $\varepsilon_{L}\;$is the complex permittivity of liver, assumed to be homogenous and with the properties of liver tissue at 2.45 GHz, as taken from the literature [28].

ΔS is the $[N_{f}\times N_{m}]\times 1$ column vector of the (complex) differential data, given by the difference between the scattering parameter measured after ablation and the scattering parameter measured before ablation with $N_{f}=201\;$being the number of frequency points and $N_{m}=13\;$the number of antenna positions. The differences between the measured S-parameters are directly fed into the inversion algorithm, without any scaling or calibration.

$K_{R}^{+}$ is the regularized pseudo inverse of the kernel matrix K, which, in the adopted model, is an M×P matrix, whose rows are ordered according to ΔS and whose generic entry $K_{mp}$ is given by

(3) $$K_{mp}=-\frac{j}{2}\pi k_{f}\rho J_{1}\left(k_{f}\rho \right)H_{0}^{2}\left(k_{f}\left|r_{q}-r_{p}\right|\right)H_{0}^{2}\left(k_{f}\left|r_{q}-r_{p}\right|\right)$$

where $f=1,\ldots N_{f};\;q=1,\ldots N_{m};p=1,\ldots ,\;P$; ρ is the radius of a circle having the same area of the pixel, $J_{1}$ is the first order Bessel function, $H_{0}^{2}$ denotes the 0-th order second kind Hankel function, $r_{q}\;$is the *q*-th position of the antenna and $r_{p}$ denotes the position of the *p*-th pixel, $k_{f}=\omega_{f}\sqrt{\varepsilon_{L}\mu_{o}}$ is the (complex) wavenumber in liver at the *f*-th pulsation $\omega_{f}$, $\mu_{o}$ is the magnetic permeability in vacuum.

To obtain $K_{T}^{+}$, let us introduce the singular value decomposition (SVD) of K, defined as

(4) $$K=U\;\Sigma \;V^{T}$$

where U is the M×M matrix whose columns are the left singular vectors (which span the space of differential data), V is the P×P matrix whose columns are the right singular vectors (which span the space of visible contrast functions) and Σ is the M×P matrix of the singular values, whose elements are all zeros but for those lying on the diagonal of the M×M submatrix (being in our case M<P). These scalars, say $s_{1},\ldots ,s_{M}\;$are ordered in decreasing fashion and accumulate to zero, that is $s_{1}>s_{2}\ldots ,s_{M-1}>s_{M}$, with $s_{n}\to 0,\;n\to \infty$.

Due to the unavoidable presence of noise on data, the direct inversion of K is unstable, since the exponentially fast growth of $1/s_{n}\;$for increasing values of n results in an uncontrolled amplification of noise. To overcome this drawback, the regularized pseudo inverse $K_{R}^{+}$ is introduced by truncating the singular value decomposition (SVD) to the first R values, with R<M, thus obtaining

(5) $$K_{R}^{+}=V_{R}\;\Sigma_{R}^{-1}U_{R}^{T}$$

where $U_{R}^{T}$ is the M×R matrix whose columns are the first R left singular vectors, $V_{R}$ is the P×R matrix whose columns are the first R right singular vectors and $\Sigma_{R}^{-1}$ is the R×R diagonal matrix, whose elements are the inverse of the first R singular values, $1/s_{i}\;i=1,\ldots ,R$.

Note that the SVD (4) is computed off-line (and only once), so that the solution (1) is achieved in real-time with a standard laptop, since it only involves (a few) matrix vector operations.

#### 2.4.1. Choice of the Regularization Parameter R

The truncation index *R* is the regularization parameter of the TSVD algorithm, and represents a degree of freedom in its implementation. In particular, a threshold as large as possible is in principle desirable to improve the accuracy. On the other hand, as mentioned before, this may induce an error amplification effect. As such, the threshold *R* is chosen as a trade-off between accuracy and stability.

**Figure 5:**
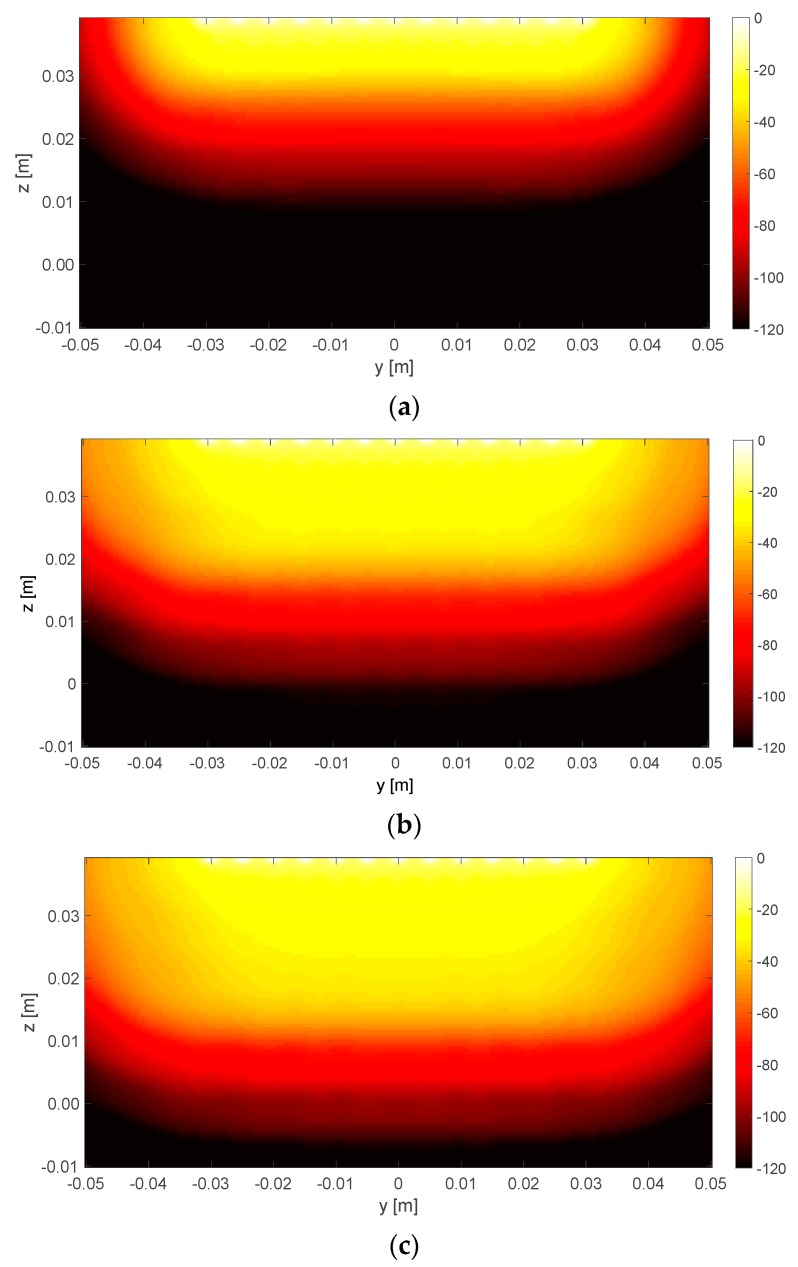
Spatial coverage of the operator $K_{R}^{+}$ for *R* equal to (**a**) 20; (**b**) 48; and (**c**) 60. In the study, *R* = 48 was set. Refer to Figure 2 for the reference system.

For the considered measurement configuration, *R* also affects the maximum depth of visible targets. Such a circumstance can be appreciated from the spatial coverage of $K_{R}^{+}$, defined as the squared amplitude of the elements of matrix $V_{R}$ summed along the columns and rearranged on the $N_{y}\times N_{z}$ grid. Figure 5 shows the spatial coverage of $K_{R}^{+}$ for different values of *R* in the yz plane at x = 0 (see Figure 2 for the reference system). In the adopted colormap, the black regions represent those portions of the imaging domain that are expected to be poorly retrieved. As can be observed, the larger the threshold, the larger the portion of the domain which is ‘covered’ by the imaging algorithm. In particular, it can be noted that R=20 only allows imaging the shallow part of the specimen. Considering that the applicator is positioned in the origin of the reference system and that an ablation zone in the order of a few centimeters is typically dealt with, the threshold was set to R=48, as this value allows imaging a sufficiently deep portion of the specimen, while keeping the number of unknown lower than R=60, which is helpful to ensure a stable result in the unavoidable presence of noise.

#### 2.4.2. Assessment Criterion

To assess the obtained imaging results, the ex-post visual inspection of the ablated specimen was exploited to build reference images to be compared with the one obtained from the processing of the experimental (differential) data. In particular, the observed size of the treated region, together with the expected values of the dielectric properties of ablated tissue [36] were used to build a 2D reference differential contrast $\Delta \chi_{ref}$. Then, the ideal imaging result is given by the projection onto the first *R* = 48 right singular functions, computed as

(6) $$\Delta \chi_{id}=V_{R}V_{R}^{T}\Delta \chi_{ref}.$$

Equation (6) provides the ideal output of the adopted imaging procedure in the considered conditions.

## 3. Results

### 3.1. Ex Vivo Post-Ablation Analysis

At the end of the MTA procedure, the specimens were sectioned along the *xy* plane, displaying the coagulative necrosis for visual inspection, and the characteristic dimensions of the zone of ablation were measured.

**Figure 6:**
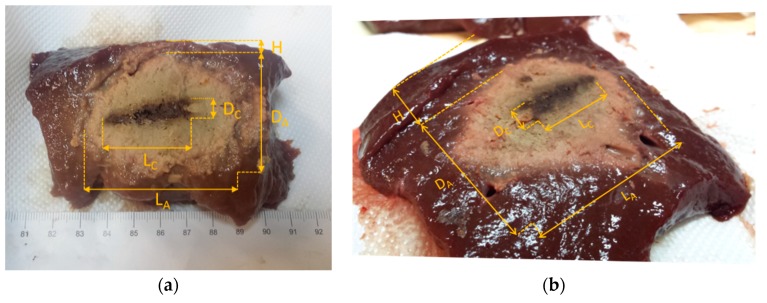
Characteristic dimensions of the zone of ablation in a coronal plane as appraised from the visual inspection. (**a**) Vivaldi experiment. (**b**) Half-heart experiment. The yellow dotted lines denote the position of interfaces of interest, while the two-way arrows identified distances.

Figure 6 shows the sectioned specimens from the two experiments with the relevant measurement superimposed. In particular, L_A_ (mm) and D_A_ (mm) represent the maximum extension of the ablated zone in the longitudinal (i.e., the length) and transverse (i.e., the diameter) directions, respectively. Likewise, L_C_ (mm) and D_C_ (mm) represent the maximum extension of the central carbonized zone in the longitudinal and transverse directions, respectively. H (mm) represents the distance between the upper boundary of the ablated zone and the surface of the tissue specimen measured post-ablation, which is the upper margin of untreated tissue in the transverse direction.

As it was shown in previous works [35], ablation-induced tissue deformation is highly heterogeneous and eventually not predictable, resulting in both tissue shrinkage and expansion, owing to interactions between the contracting thermally-coagulated tissue and the untreated tissue encompassing the zone of ablation, as well as to expansion of water steam diffusing from the inner zone of ablation. As discussed in the following, this aspect represents a non-trivial issue in the assessment pursued in this work.

In Table 1, the characteristic dimensions of the zone of ablation are summarized, along with the net power (mean value ± standard deviation, W) supplied to the applicator during the MTA procedure. From the reported data, it is apparent that in both MTA experiments tissue specimens showed ablation-induced deformation in the transverse direction (D_A_/2 + H − 30, being 30 mm the distance pre-ablation between the applicator and the surface of the specimen). Specifically, in the experiment with the Vivaldi antenna the specimen was characterized by a contraction of about 7 mm, whereas in the half-heart antenna case, the specimen exhibited an expansion of about 5 mm. Such an outcome cannot be easily foreseen or modeled. As an ex-post observation, it can be noted that, as summarized in Table 1, the experiment with the half-heart antenna lead to a larger transverse ablation diameter (D_A_) with respect to the one in the experiment with the Vivaldi antenna, i.e., 44 mm vs. 38 mm. Therefore, it can be argued that the more superficial ablation achieved in the experiment with the half-heart antenna may have facilitated the upwards propagation of vapor gases, thus causing tissue transverse expansion. However, such an outcome cannot be generalized, since the ultimate result of an ablation procedure (and then of ablation-induced tissue deformation) relies on heat propagation, which is also affected by tissue morphology around the applicator (e.g., presence of small blood vessels or local non-homogeneities).

### 3.2. Microwave Tomography Results

The expansion of the liver sample observed in the post-ablation visual inspection in the case of half-hearth experiment confirmed a difficulty occurred when performing the MWT measurements with the antenna in contact with the liver. As a matter of fact, due to the swelling, the antenna was somehow ‘immersed’ in liver in some positions. For this reason, the relevant dataset was excluded from the tomographic processing.

**Figure 7:**
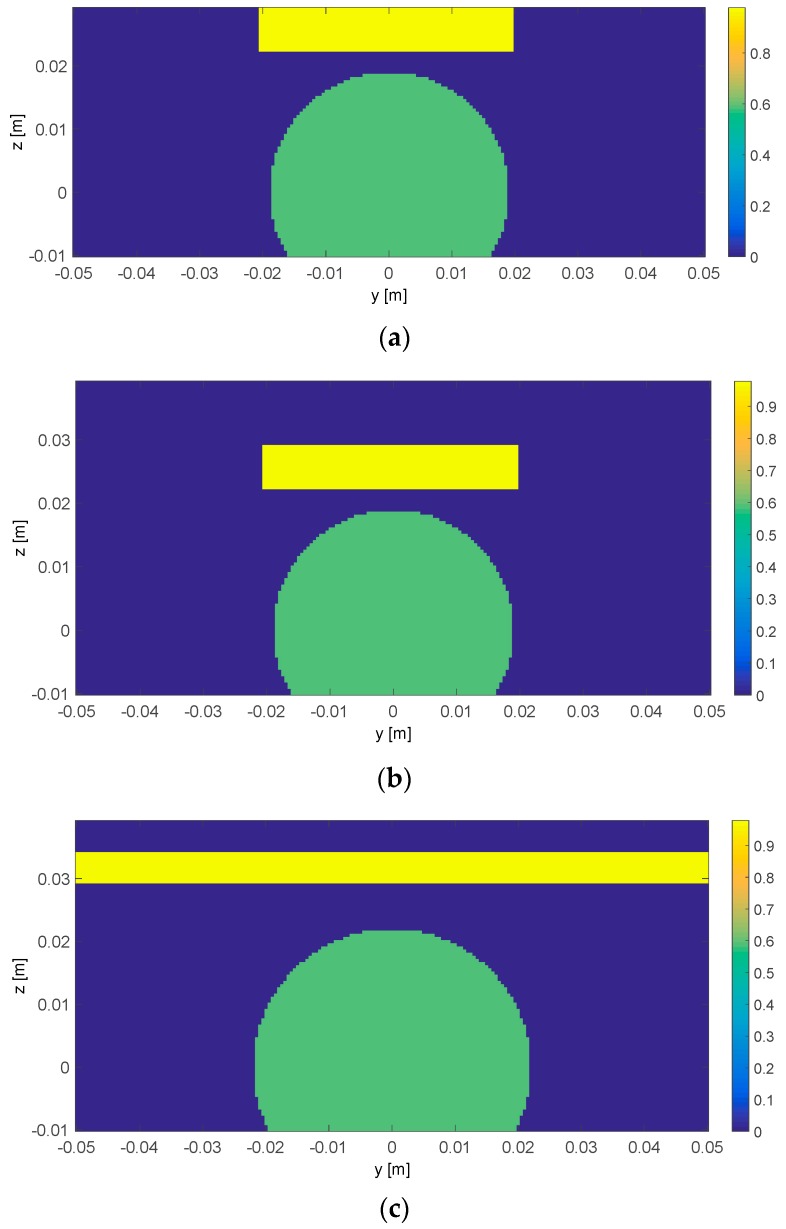
Ideal differential contrast for the analyzed cases. Each figure represents the *yz* cross-section of the ground truth as deduced from visual inspection. The green areas are ablated tissue, the yellow areas are specimen surface modifications. The MTA applicator is located at (0 m, 0 m). The MWT antenna moves along the top border of each figure. (**a**) Vivaldi experiment with antenna in contact; (**b**) Vivaldi experiment with antenna at 10 mm from the specimen; (**c**) Half-heart experiment with antenna at 10 mm from the specimen. The bar chart of each sub-figure are in the same scale and they denote the absolute value of the differential contrast for the three analyzed cases.

For the remaining three available datasets, Figure 7 shows the ideal contrast functions $\Delta \chi_{ref}$ according to the visual analysis of the post-ablation liver specimens described in the previous section. In these images, the variations between the two states are evidenced: the green areas identify the ablated tissue, whereas the yellow areas correspond to modifications of the specimen caused by shrinkage in the Vivaldi experiment and swelling in half-heart case. Of course, given the heterogeneous and not predictable nature of tissue ablation and deformation, these images cannot provide an accurate model of the ground truth, nevertheless, they retain the features which are mostly relevant for the imaging task.

**Figure 8:**
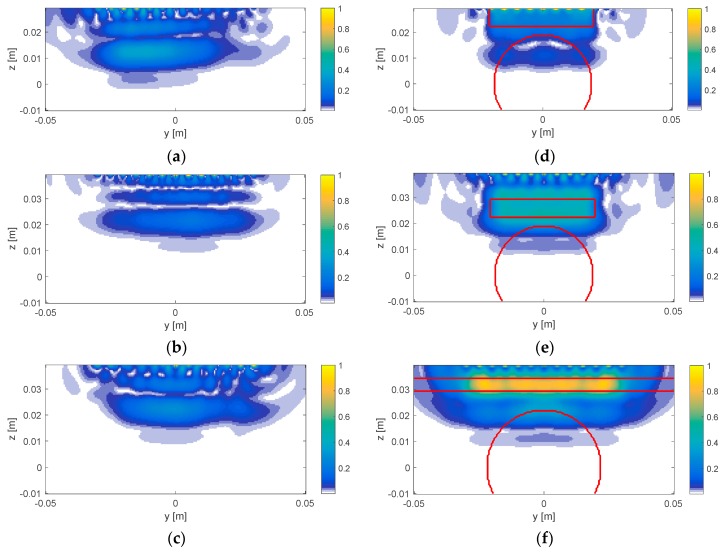
Comparison between tomographic results and ideal reconstructions. Each figure reports the plot of a cross-section in the *yz* plane of the retrieved contrast (normalized modulus). In the images, the main features (in terms of qualitative variations) of the differential contrast appear. Left side, tomographic images: (**a**) Vivaldi experiment with antenna in contact with liver sample; (**b**) Vivaldi experiment with antenna at 1 cm from sample; (**c**) Half-heart experiment with antenna at 1 cm height. Right side, (**d**–**f**) ideal reconstructions. Red contours denote the contour of the ideal differential contrast, as identified in Figure 7. All mps are normalized to their maximum. As such, colorbars range from 0 to 1. 0 values represent areas where no variation occurrs, while 1 values denote areas where the maximum variation is observed.

In Figure 8, the imaging results for the processed datasets are reported in terms of the normalized amplitudes of the estimated differential contrast, i.e., |Δχ| for the tomographic images and $\left|\Delta \chi_{id}\right|$ for the ideal reconstructions. As a matter of fact, while the algorithm actually provides an estimate of the (complex) differential contrast Δχ, the very limited amount of available data and the aspect limited nature of the measurement configuration, which prevents a complete estimate of the unknown function, do not allow retrieving the actual quantitative values. For this reason, the images are given in terms of the retrieved differential contrast amplitude, as this provides a qualitative estimate of the main variations occurring in the imaged zone. As said, such information corresponds to the first clinically relevant goal to achieve.

In the figure, the left column shows the tomographic image obtained from processing the experimental data, whereas the right column reports the ideal reconstructions, obtained by applying Equation (6) to the differential contrast of the relevant reference scenario shown in Figure 7. On the images on the right of the figure, the contour of the ablated area and the surface modifications are superimposed to facilitate interpretation. To allow better appraisal of the results, taking into account the differential nature of the imaging approach, the adopted color bar forces the false colors to white in the areas where the amplitude of the differential contrast is estimated as zero. It is worth noting that obtaining these images requires less than 1 s on a standard desktop pc.

## 4. Discussion

The comparison between the tomographic images and the ideal images allowed both to interpret the results as well as to appraise their quality and the open issues that emerged from the present study.

The ablated specimens underwent significant surface changes during the thermal treatment. In a differential imaging framework, such modifications are retrieved by the algorithm and appear as ‘targets’ in the images. As such, the obtained images will not just report the contrast variations due to the ablation of the tissue, but also the variations related to the post-ablation specimen deformation. Such a circumstance is confirmed by the observation of the ideal images in the right column of Figure 8, wherein the deformation is clearly visible. In the tomographic results, such features are also visible and are correctly positioned.

As far as the transition between treated and untreated tissue is concerned, in the Vivaldi experiment, the separation between the surface of the sample and the ablated tissue (i.e., the margin of untreated tissue in the *z* direction) is lower than 0.5 cm, due ablation-induced shrinkage of the specimen. Such a distance is in the order or even below the expected spatial resolution of MWT at the adopted frequency band ($\delta =\frac{C}{2B}$, with *c* being the velocity of propagation in the liver and *B* the bandwidth of the signal), which—for the case at hand—is about 8 mm. Accordingly, the experimental conditions did not allow to appreciate the separation between untreated (and shrunk) tissue and treated tissue. This is confirmed by the observation of the ideal images, wherein indeed the position of the treated-to-ablated tissue boundary cannot be discriminated. Nevertheless, despite this limitation, the tomographic images obtained from the experimental data are consistent with the ideal ones.

For the half-heart case, the swelling of the specimen had an opposite effect, so that no overlap is expected and the transition from treated to untreated tissue is expected to be properly imaged, as confirmed by the ideal images. This is fully confirmed by the experimental result, wherein both the effect of the swelling and the treated-to-untreated boundary are correctly imaged. Besides, a good agreement with the ideal results is obtained.

Accordingly, a first experimental evidence of the capability of MWT of detecting the transition between treated and untreated tissue was achieved, using a procedure which can be easily implemented in real time. This is indeed one of the main clinical goals to pursue, to propose a new technique for real-time monitoring the evolution of thermal ablation treatment.

## 5. Conclusions

In this paper, the outcomes of the first experimental proof-of-concept of thermal ablation monitoring via MWT were presented. The study aimed at assessing the feasibility of MWT as a real-time monitoring tool for thermal ablation treatments. To this end, a laboratory set-up was designed to set an *ex vivo* experiment in which the changes in the electromagnetic properties of two bovine liver specimens where imaged by means of MWT measurement and processing. Overall, the results confirmed the anticipated potential of MWT, but a number of interesting issues aroused, which deserve further investigation in future research work.

The main drawback occurred due to the modification of the specimen volume during ablation. Although this effect was to some extent expected in *ex vivo* experiments, whether it actually occurs in vivo or in clinical situations is not clear. In fact, the liver-air system herein dealt with is a much simpler scenario from a thermodynamic point of view than the actual scenario, wherein the treated region is surrounded by the parenchyma or other biological tissue. To partially cope with this issue, and also move to a more complex *ex vivo* scenario, future experiments will deal with a three-layer structure, in which a matching medium (whose properties have to be properly chosen) is positioned between the antennas and the liver specimen. This layer may both mimic the tissue surrounding the liver and provide a surrounding medium which may absorb the tissue deformation. In addition to this, the use of a matching medium can improve the performance of the antennas, which in turn can reduce uncertainty on data and therefore allow inspecting more in depth. To this end, the design of ad hoc antennas is of course a crucial aspect, since the antennas used in this study provide sub-optimal, yet acceptable, performances in the measurement frequency band.

The presented experiments aimed at pursuing the initial goal of observing and assessing the two extreme cases of non-ablated and ablated tissue. Their positive outcome stimulates a campaign of experiments that will address the monitoring of an ongoing treatment, by performing measurements at intermediate ablation stages also. In this respect, the need of coping with measurements taken during the operation of the thermal applicator represents an interesting issue to investigate, not only to understand the effect of the MW heating signal onto the measured data (possible interference), but also to examine the possible cooperative role of the applicator as well as the possibility of devising interleaved treatment/measurement protocols in which the thermal ablation and the monitoring are performed alternatively.

Finally, performing a number of linear scans along parallel rectilinear paths can be foreseen as way to gather a sufficient amount of data in order to build more accurate, possibly 3D maps of the monitored scenario. In this respect, the use of an array of antennas could be envisaged in order to keep the measurement time as low as possible, by resorting to electronic rather than mechanical scanning.

## Figures and Tables

**Table 1:** Characteristic dimensions of the ablation zone achieved in the MTA experiments

MWT Antenna	Power (W)	L_A_ (mm)	D_A_ (mm)	L_C_ (mm)	D_C_ (mm)	H (mm)	Remarks
Vivaldi	54.8 ± 0.8	56	38	36	8	4	transverse contraction ~7 mm
Half-heart	57.8 ± 1.2	54	44	31	8	13	transverse expansion ~5 mm

**Figure A1:**
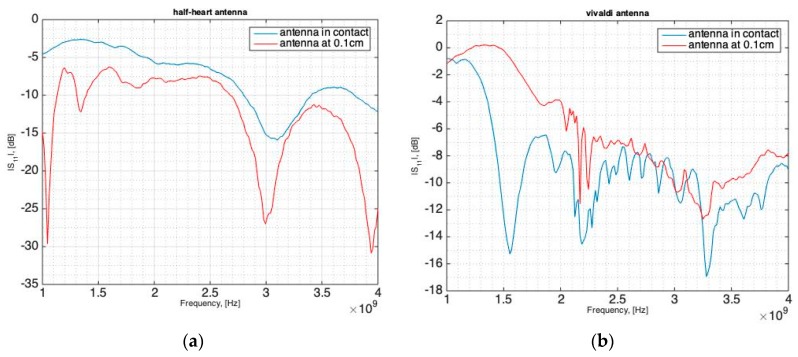
Amplitude of the S_11_ scattering parameters as measured for the antenna in the central position (in correspondence of the applicator): (**a**) Half-heart antenna. (**b**) Vivaldi antenna.

## Appendix A

In this appendix, the measured scattering parameters (S_11_) for the two antennas adopted in the experiment are reported.

Figure A1 shows the scattering parameter measured with the antenna located in correspondence of the applicator axis (*y* = 0) in contact with the liver tissue or placed at 1 cm distance from the surface of the liver. In particular, Figure A1a reports the data for the half-heart antenna, while Figure A1b shows the data related to the Vivaldi antenna.

As can be seen, despite the antennas were not designed for this particular application, the value of the S_11_ parameter (on average) in the considered frequency band is below −5 dB which is quite acceptable. In addition, a better performance is observed for the half-hearth antenna trough out the whole band.

## Appendix B

This appendix provides the formulation of the inverse scattering method adopted to process the data gathered in the experimental study.

The input data is given by the difference between scattering parameters collected by each antenna before and after ablation. Hence, for each of the 201 frequency points in the measured bandwidth and for each of the 13 antenna positions, a differential scattering parameter, say S, is obtained.

For a given position *r_x_* of the MWT antenna and for each pulsation ω in the measurement bandwidth, the differential scattering parameter can be expressed as

(A1) $$\Delta S=S_{11}^{a}-S_{11}^{0}=K\left[\Delta \chi \right]$$

where $S_{11}^{a}$ is the scattering parameter measured after ablation and $S_{11}^{0}$ is the scattering parameter measured before ablation, respectively.

Δχ is the differential contrast, which encodes the changes occurring in the electromagnetic properties of the specimen due to the ablation. Such a function is defined as

(A2) $$\Delta \chi \left(r,\omega \right)=\frac{\varepsilon_{a}\left(r,\omega \right)}{\varepsilon_{L}\left(r,\omega \right)}-1$$

where *r* = (*x,y*) denotes the generic position in the imaged region Ω, $\varepsilon_{a}$ is the complex permittivity profile in Ω after the ablation and $\varepsilon_{L}\;$is the complex permittivity profile in the liver specimen before ablation, which is assumed as homogeneous, with the same properties as liver tissue at 2.45 GHz. K is the short notation for the integral operator encoding the imaging kernel, whose explicit expression is given by

(A3) $$K\left[\Delta \chi \right]=\int_{\Omega }G\left(k,r_{x},r\right)G\left(k_{f},r,r_{x}\right)\Delta \chi \left(r,\omega \right)dr$$

where $r_{x}$ is the position of the antenna, $k=\omega \sqrt{\varepsilon_{L}\mu_{o}}$ is the (complex) wavenumber in liver at the working frequency and G is the Green’s function for the unperturbed scenario, that is the solution of the Helmholtz equation

(A4) $$\nabla^{2}G\left(r,r_{x}\right)-k^{2}G\left(r,r_{x}\right)=-j\omega \mu_{o}\;\delta \left(r-r_{x}\right),$$

with $G\left(r,r_{x}\right)=G\left(r_{x},r\right)$ by virtue of reciprocity. The Born approximation is adopted to cast (A4), since the Green function both models the radiation of the induced current to the receiving antenna, $G\left(r_{x},r\right)$, as well as the field induced by the antenna in the region of interest, $G\left(r,r_{x}\right)$.

In our approach, the Green function is simply modeled as the Green function pertaining to an unbounded homogeneous medium having the properties of liver, which reads

(A5) $$G\left(r,r_{x}\right)=H_{0}^{2}\left(k\left|r-r_{x}\right|\right),$$

with $H_{0}^{2}$ denoting the 0th order second kind Hankel function. Of course, more sophisticated modeling could be adopted by using for instance a numerical model of the actual antennas placed on the specimen-filled box, but a simpler model is intrinsically more robust against uncertainties arising in the actual scenario (e.g., actual shape of the sample, actual radiation pattern of antenna in presence of the specimen).

The above model provides the basis for the equation to be inverted. In particular, by discretizing Ω into $N_{x}\times N_{y}$ square pixels and gathering all the differential data into a $[N_{f}\times N_{m}]\times 1$ column vector ΔS, with $N_{f}\;$being the number of frequency points and $N_{m}\;$the number of antenna positions, the discretized data-to-unknown relationship that has to be inverted is given by

(A6) ΔS=K Δχ
